# Supplementary material for: Heat adaptation of phage T7 under an extended genetic code
Source: Virus Evol. 2021 Dec 1;7(2):veab100. doi: 10.1093/ve/veab100 (PMC8923235; doi:10.1093/ve/veab100)
Supplement: veab100_Supp [file veab100_supp.zip › Sequencing_Analysis.zip!/Sequencing_Analysis/S1/evidence/SNP_13.html]

BRESEQ :: Evidence


| Predicted mutation | | | | | | |
| --- | --- | --- | --- | --- | --- | --- |
| evidence | seq id | position | mutation | annotation | gene | description |
| RA | NC\_001604.1 | 37,346 | T→C | intergenic (–/–) | – / – | –/– |

| Read alignment evidence... | | | | | | | | | | | |
| --- | --- | --- | --- | --- | --- | --- | --- | --- | --- | --- | --- |
|  | seq id | position | | ref | new | freq | score (cons/poly) | reads | annotation | genes | product |
| \* | NC\_001604.1 | 37,346 | 0 | T | C | 99.0% | 12892.0 / NA | 4284 | intergenic (–/–) | *–/–* | –/– |
| Reads supporting (aligned to +/- strand):  **ref** base T (6/7);  **major** base C (2030/2190);  **minor** base A (15/26);  **total** (2055/2229) | | | | | | | | | | | |
| Fisher's exact test for biased strand distribution *p*-value = 1.59e-01 | | | | | | | | | | | |
| Kolmogorov-Smirnov test that lower quality scores support variant *p*-value = 1.00e+00 | | | | | | | | | | | |

Only 100 of 4284 total aligned reads displayed.  

`AGTTGAGGCTGCGAAGAGCACTCAAAGAGCAATCGATGCGGTATCTGCTAAGTATCAAGAAGACCTTGCCGCGCTGGAAGGGAGCACTGATAGGATTATTTCTGATTTGCGTAGCGACAATAAGCGGTTGCGCGTCAGAGTCAAAACTACCGGAACCTCCGATGGTCAGTGTGGATTCGAGCCTGATGGTCGAGCCGAACTTGACGACCGAGATGCTAAACGTATTCTCGCAGTGACCCAGAAGGGTGACGCATGGATTCGTGCGTTACAGGATACTATTCGTGAACTGCAACGTAAGTAGGAAATCAAGTAAGGAGGCAATGTGTCTACTCAATCCAATCGTAATGCGCTCGTAGTGGCGCAACTGAAAGGAGACTTCGTGGCGTTCCTATTCGTCTTATGGAAGGCGCTAAACCTACCGGTGCCCACTAAGTGTCAGATTGACATGGCTAAGGTGCTGGCGAATGGAGACAACAAGAAGTTCATCTTACAGGCTTTCCGTGGTATCGGTAAGTCGTTCATCACATGTGCGTTCGTTGTGTGGTCCTTATGGAGAGACCCTCAGTTGAAGATACTTATCGTATCAGCCTCTAAGG  >  NC_001604.1/37048‑37643`  

                                                                                                                                                                                                                                                                                                          `|`                                                                                                                                                                                                                                                                                                           
`agttgaagcttgcatgagagcTCAAAGAGCAATCGATTCGGTATCTGCTAAGAATCAAGAAGACCTAGCCTCGATGGAAGGTAGCACTGGTAGGATTATTTCTGATTTGCATAGCGACAATAAGCTGTTGGGCGTCAGAGTCAAAACTACCGGAACCTCCGATGGTCAGTGTGGATTCGAGCCTGATGGTCGAGCCGAACTTGACGACCGAGATGCTAAACGTATTCTCGCAGTGACCCAGAAGGGTGACGCATGGATTCGTGCGTTACAGGATACTATTCGTGAACTGCAACGTAAGCAg                                                                                                                                                                                                                                                                                                         <  1:167258/281‑1 (MQ=255)`  
          `gCGAAGAGCACTCAAAGAGCAATCGATGCGGTATCTGCTAAGTATCAAGAAGACCTTGCCGCGCTGGAAGGGAGCACTGATAGGATTATTTCTGATTTGCGTAGCGACAATAAGCGGTTGCGCGTCAGAGTCAAAACTACCGGAACCTCCGATGGTCAGTGTGGCTTAGTGCCTGATGGTCGAGCCGAACTTGACGACCGAGATGCTAAACGTATTCTCGCAGTGACCCAGACGGGTGACGCATGGATTCGTGCGTTACAGGATACTATTCGTGACCTGCAACGTAAGCAGGAAATCAAGt                                                                                                                                                                                                                                                                                               >  1:488724/1‑301 (MQ=255)`  
            `gAAGAGCACTCAAAGAGCAATCGATGCGGTATCTGCTAAGTATCAAGAAGCCCTTGCCGCGCTGGAAGGGAGCACTGATAGGATTATTTCTGATTTGCGTAGCGACAATAAGCGGTTGCGCGTCAGAGTCAAAACTACCGGAACCTCCGATGGTCAGTGTGGATTCGAGCCTGATGGTCGAGCCGAACTTGACGACCGAGATGCTAAACGTATTCTCGCAGTGACCCAGAAGGGTGACGCATGGATTCGTGCGTTACAGGATACTATTCGTGAACTGCAACGTAAGCAGGAAATCAAGTaa                                                                                                                                                                                                                                                                                             <  1:371430/301‑1 (MQ=255)`  
              `agggCACTCAAAGAGCAAGCGATGCGGTATCTGCTAAGTATCAAGAAGACCTTGCCGCGCTGGAAGGGAGCACTGGTAGGATTATTTCTGATTTGCGTAGCGACAATAAGCGGGTGCGCGTCAGAGTCAAAACTACCGGAACCTCCGATGGTCAGTGTGGATTCGAGCCTGATGGTCGAGCCGAACTTGACGACCGAGATGCTAAACGTATTCTCGCAGTGACCCAGAAGGGTGACGCATGGATTCGTGCGTTACAGGATACTATTCGTGAACTGCAACGTAAGCAGGAAATCAAGTAagg                                                                                                                                                                                                                                                                                           <  1:510653/298‑1 (MQ=255)`  
               `gagCACTCAAAGAGCAATCGATGCGGTATCTGCTAAGTATCAAGAAGACCTTGCCGCGCTGGAAGGGAGCACTGATAGGATTATTTCTGATTTGCGTAGCGACAATAAGCGGTTGAGCGTCAGAGTCAAAACTACCGGAACCTCCGATGGTCAGTGTGGATTCGAGCTTGATGGTCGAGCCGAACTTGACGACCGAGATGCTAAACGTATTCTCGCAGTGACCCAGAAGGGTGACGCATGGATTCGTGCGTTACAGGATACTATTCGTGAACTGCAACGTAAGCAGGAAATCAAGTAagga                                                                                                                                                                                                                                                                                          >  1:271127/1‑301 (MQ=255)`  
                    `cTCAAAGAGCAATCGATGCGGTATCTGCTAAGTATCAAGAAGACCTTGCCGCGCTGGAAGGGAGCACTGATAGGATTATTTCTGATTTGCGTAGCGACAATAAGCGGTTGCGCGTCAGAGTCAAAACTACCGGAACCTCCGATGGTCAGTGTGGATTCGAGCCTGATGGTCGAGCCGAACTTGACGACCGAGATGCTAAACGTATTCTCGCAGTGACCCAGAAGGGTGACGCATGGATTCGTGCGTTACAGGATACTATTCGTGAACTGCAACGTAAGCAGGAAATCAAGTAAGGAGGCaa                                                                                                                                                                                                                                                                                     >  1:11827/1‑301 (MQ=255)`  
                      `aaaaGAGCAATCGATGCGGTATCTGCTAAGTATCAAGAAGACCTTGCCGCGCTGGAAGGGAGCACTGATAGGATTATTTCTGATTTGCGTAGCGACAATAAGCGGTTGCGCGTCAGAGTCAAAACTACCGGAACCTCCGATGGTCAGTGTGGATTCGAGCCTGATGGTCGAGCCGAACTTGACGACCGAGATGCTAAACGTATTCTCGCAGTGACCCAGAAGGGTGACGCATGGATTCGTGCGTTACAGGATACTATTCGTGAACTGCAACGTAAGCAGGAAATCAAGTAAGGAGGCAAtg                                                                                                                                                                                                                                                                                   <  1:337873/300‑1 (MQ=255)`  
                       `aaaGAGCAATCGATGCGGTATCTGCTAAGTATCAAGAAGACCTTGCCGCGCTGGAAGGGAGCACTGATAGGATTATTTCTGATTTGCGTAGCGACAATAAGCGGTTGCGCGTCAGAGTCAAAACTACCGGAACCTCCGATGGTCAGTGTGGATTCGAGCCTGATGGTCGAGCCGAACTTGACGACCGAGATGCTAAACGTATTCTCGCAGTGACCCAGAAGGGTGACGCATGGATTCGTGCGTTACAGGATACTATTCGTGAACTGCAACGTAAGCAGGAAATCAAGTAAGGAGGACAtgt                                                                                                                                                                                                                                                                                  >  1:46356/1‑301 (MQ=255)`  
                        `aaGAGCAATCGATGCGGTATCTGCTAAGTATCCAGAAGACCTTGCCGCGCTGGAAGGGAGCACTGATAGGATTATTTCTGATTTGCGTAGCGACAATAAGCGGTTGCGCGTCAGAGTCAAAACTACCGGAACCTCCGATGGTCAGTGTGGATTCGAGCCTGATGGTCGAGCCGAACTTGACGACCGAGATGCTAAACGTATTCTCGCAGTGACCCAGAAGGGTGACGCATGGATTCGTGCGTTACAGGATACTATTCGTGAACTGCAACGTAAGCAGGAAATCAAGTAAGGAGGCAAtgtg                                                                                                                                                                                                                                                                                 <  1:202644/301‑1 (MQ=255)`  
                          `gagCAATCGATGCGGTATCTGCTAAGTATCAAGAAGACCTTGCCGCGCTGGAAGGGAGCACTGATAGGATTATTTCTGATTTGCGTAGCGACAATAAGCGGTTGCGCGTCAGAGTCAAAACTACCGGAACCTCCGATGGTCAGTGTTGATTCGAGCCTGATGGTCGAGCCGAACTTGACGACCGAGTTGCTAAACGTATTCTCGCAGTGACCCAGACGGGTGACGTATGGATTCGTCCGTTACAGGATACTATTCGTGAACTGCAACGTAAGCCGGAAATCAAGTAAGGAGGCAAtgtctt                                                                                                                                                                                                                                                                               >  1:529307/1‑298 (MQ=255)`  
                          `gagCAATCGATGCGGTATCTGCTAAGTATCAAGAAGACCTTGCCGCGCTGGAAGGGAGCACTGATAGGATTATTTCTGATTTGCGTAGCGACAATAAGCGGTTGCGCGTAAGAGTCAAAACTACCGGAACCTCCGATGGTCAGTGTGGATTCGAGCCTGATGGTCGAGCCGAACTTGACGACCGAGATGCTAAACGTATTCTCGCAGTGACCCAGAAGGGTGACGCATGGATTCGTGCGTTACAGGATACTATTCGTGAACTGCCACGTAAGCAGGAAATCAAGTAAGGAGGCAATgtgtt                                                                                                                                                                                                                                                                               >  1:445680/1‑300 (MQ=255)`  
                             `cAATCGATGCGGTATCTGCTAAGTATCAAGAAGACCTTGCCGCGCTGGAAGGGAGCACTGATAGGATTATTTCTGATTTGCGTAGCGACAATAAGCGGTTGCGCGTCAGAGTCAAAACTACCGGAACCTCCGATGGTCAGTGTGGATTCGAGCCTGATGGTCGAGCCGAACTTGACGACCGAGATGCTAAACGTATTCTCGCAGTGACCCAGAAGGGTGACGCATGGATTCGTGCGTTACAGGATACTATTCGTGAACTGCAACGTAAGCAGGAAATCAAGTAAGGAGGCAATGTGTCTAc                                                                                                                                                                                                                                                                            <  1:562639/301‑1 (MQ=255)`  
                             `aaaTCGATTCGGTATCTGCTAAGTATCAAGAAGACCTTGCCGCGCTGGAAGGGAGCACTGATAGGATTATTTCTGATTTGCGTAGCGACAATAAGCGGTTGCGCGTCAGAGTCAAAACTACCGGAACCTCCGATGGTCAGTGTGGATTCGAGCCTGATGGTCGAGCCGAACTTGACGACCGAGATGCTAAACGTATTCTCGCAGTGACCCAGAAGGGTGACGCATGGATTCGTGCGTTACAGGATACTATTCGTGAACTGCAACGTAAGCAGGAAATCAAGTAAGGAGGCAATGTGTCTAc                                                                                                                                                                                                                                                                            <  1:5457/300‑1 (MQ=255)`  
                               `aTCGATGCGGTATCTGCTAAGTATCAAGAAGACCTTGCCGCGCTGGAAGGGAGCACTGATAGGATTATTTCTGATTTGCGTAGCGACAATAAGCGGTTGCGCGTCAGAGTCAAAACTACCGGAACCTCCGATGGTCAGTGTGGATTCGAGCCTGATGGTCGAGCCGAACTTGACGACCGAGATGCTAAACGTATTCTCGCAGTGACCCAGAAGGGTGACGCATGGATTCGTGCGTTACAGGATACTATTCGTGAACTGCAACGTAAGCAGGAAATCAAGTAAGGAGGCAATGTGTCTACTc                                                                                                                                                                                                                                                                          <  1:13371/301‑1 (MQ=255)`  
                               `aTCGATGCGGTATCTGCAAAGTATCAAGAAGACCTTGCCGCGCTGGAAGGGAGCACTGATAGGATTATTTCTGATTTGCGTAGCGACAATAAGCGGTTGCGCGTCAGAGTCAAAACTACCGGAACCTCCGATGGTCAGTGTGGATTCGAGCCTGATGGTCGAGCCGAACTTGACGACCGAGATGCTAAACGTATTCTCGCAGTGACCCAGAAGGGTGACGCATGGATTCGTGCGTTACAGGATACTATTCGTGAACTGCAACGTAAGCAGGAAATCAAGTAAGGAGGCAATGTGTCTACTc                                                                                                                                                                                                                                                                          <  1:504934/301‑1 (MQ=255)`  
                                   `aTGCGGTATCTGCTAAGTATCAAGAAGACCTTGCCGCGCTGGAAGGGAGCACTGATAGGATTATTTCTGATTTGCGTAGCGACAATAAGCGGTTGCGCGTCAGAGTCAAAACTACCGGAACCTCCGATGGTCAGTGTGGATTCGAGCCTGATGGTCGAGCCGAACTTGACGACCGAGATGCTAAACGTATTCTCGCAGTGACCCAGAAGGGTGACGCATGGATTCGTGCGTTACAGGATACTATTCGTGAACTGCAACGTAAGCAGGAAATCAAGTAAGGAGGCAATGTGTCTACTcctgt                                                                                                                                                                                                                                                                      >  1:191303/1‑297 (MQ=255)`  
                                   `aTGCGGTATCTGCTAAGTATCAAGAAGACCTTGCCGCGCTGGAAGGGAGCACTGATAGGATTATTTCTGATTTGCGTAGCGACAATAAGCGGTTGCGCGTCAGAGTCAAAACTACCGGAACCTCCGATGGTCAGTGTGGATACGAGCCTGATGGTCGAGCCGAACTTGACGACCGAGATGCTAAACGTATTCTCGCAGTGACCCAGAAGGGTGACGCATGGATTCGTGCGTTACAGGATACTATTCGTGAACTGCAACGTAAGCAGGAAATCAAGTAAGGAGGCAATGTGTCTACTcaatc                                                                                                                                                                                                                                                                      >  1:515469/1‑301 (MQ=255)`  
                                    `tGCGGTATCTGCTAAGTATCAAGCAGACCTTGCCGCTCTGGAAGGGAGCACTGATAGGATTATTTCTGATTTGCAGAGCGACAATAAGCGGTTGCGCGTCAGAGTCAAAACTTCCGTAACCTCCGATGGTCAGTGTTGATTCGAGCCTGATGGTCGAGCCGAAGTTGACGACCGAGATGCTAAACGTATTCTCGCAGTGACCCAGAAGGGTGACGCATGGATTCGTGCGTTACAGGATACTATTCGTGAACTGCAACGTAAGCAGGAAATCAAGTAAGGAGGCAATGTGTCTACTcaatcc                                                                                                                                                                                                                                                                     <  1:283251/301‑1 (MQ=255)`  
                                      `cGGTATCTGCTAAGTATCAAGAAGACCTTGCCGCGCTGGAAGGGAGCACTGATAGGATTATTTCTGATTTGCGTAGCGACAATAAGCGGTTGCGCGTCAGAGTCAAAACTACCGGAACCTCCGATGGTCAGTGTGGATTCGAGCCTGATGGTCGAGCCGAACTTGACGACCGAGATGCTAAACGTATTCTCGCAGTGACCCAGAAGGGTGACGCATGGATTCGTGCGTTACAGGATACTATTCGTGAACTGCAACGTAAGCAGGAAATCAAGTAAGGAGGCAATGTGTCTACTcaatccaa                                                                                                                                                                                                                                                                   >  1:72734/1‑301 (MQ=255)`  
                                       `ggTATCTGCTAAGTATCAAGAAGACCTTGCCGCGCTGGAAGGGAGCACTGATAGGATTATTTCTGATTTGCGTAGCGACAATAAGCGGTTGCGCGTCAGAGTCAAAACTACCGGAACCTCCGATGGTCAGTGTGGATTCGAGCCTGATGGTCGAGCCGAACTTGACGACCGAGATGCTAAACGTATTCTCGCAGTGACCCAGAAGGGTGACGCATGGATTCGTGCGTTACAGGATACTATTCGTGAACTGCAACGTAAGCAGGAAATCAAGTAAGGAGGCAATGTGTCTACTcaatccaat                                                                                                                                                                                                                                                                  >  1:326573/1‑301 (MQ=255)`  
                                            `cTGCTAAGTATCAAGAAGACCTTGCCGCGCTGGAAGGGAGCACTGATAGGATTATTTCTGATTTGCGTAGCGACAATAAGCGGTTGCGCGTCAGAGTCAAAACTACCGGAACCTCCGATGGTCAGTGTGGATTCGAGCCTGATGGTCGAGCCGAACTTGACGACCGAGATGCTAAACGTATTCTCGCAGTGACCCAGAAGGGTGACGCATGGATTCGTGCGTTACAGGATACTATTCGTGAACTGCAACTTAAGCAGGAAATCAAGTAAGGAGGCAATGTGTCTACTCAATCCAATCGTaa                                                                                                                                                                                                                                                             >  1:350960/1‑301 (MQ=255)`  
                                            `cTGCTAAGTATCAAGAAGACCTTGCCGCGCTGGAAGGGAGCACTGATAGGATTATTTCTGATTTGCGTAGCGACAATAAGCGGTTGCGCGTCAGAGTCAAAACTACCGGAACCTCCGATGGTCAGTGTGGATTCGAGCCTGATGGTCGAGCCGAACTTGACGACCGAGATGCTAAACGTATTCTCGCAGTGACCCAGAAGGGTGACGCATGGATTCGTGCGTTACAGGATACTATTCGTGAACTGCAACGTAAGCAGGAAATCAAGTAAGGAGGCAATGTGTCTACTcaatcctgtctctt                                                                                                                                                                                                                                                             >  1:583155/1‑293 (MQ=255)`  
                                            `cTGCTAAGTATCAAGAAGACCTTGCCGCGCTGGAAGGGAGCACTGATAGGATTATTTCTGATTTGCGTAGCGACAATAAGCGGTTGCGCGTCAGAGTCAAAACTACCGGAACCTCCGATGGTCAGTGTGGATTCGAGCCTGATGGTCGAGCCGAACTTGACGACCGAGATGCTAAACGTATTCTCGCAGTGACCCAGAAGGGTGACGCATGGATTCGTGCGTTACAGGATACTATTCGTGAACTGCAACGTAAGCAGGAAATCAAGTAAGGAGGCAATGTGTCTACTCAATCCAATCGTaa                                                                                                                                                                                                                                                             >  1:215642/1‑301 (MQ=255)`  
                                              `gCTAAGTATCAAGAAGACCTTGCCGCGCTGGAAGGGAGCACTGATAGGATTATTTCTGATTTGCGTAGCGACATTAAGCGGTTGCGCGTCAGAGTCAAAACTACCGGAACCTCCGATGGTCAGTGTGGATTCGAGCCTGATGGTCGAGCCGAACTTGACGACCGAGATGCTAAACGTATTCTCGCAGTGACCCAGAAGGGTGACGCATGGATTCGTGCGTTACAGGATACTATTCGTGAACTGCAACGTAAGCAGGAAATCAAGTAAGGAGGCACTGTGTCTACTCAATCCAATCGTAATg                                                                                                                                                                                                                                                           >  1:221212/1‑301 (MQ=255)`  
                                              `gCTAAGTATCAAGAAGACCTAGCCGCGATGGAAGGGAGCACGGATAGGATTATTTCTGATTTGCGGAGAGACAATAAGCGGTGGCGAGTCAGAGTTAAAACTACCGGAACCTCCGATGGTCAGTGTGGAGTCGAGCCTGATGGTCGAGCCGAACTGGACGACCGAGATGCTAAACGGATTCTCGCAGTGACCCAGAAGGGTGACGCATGGAGTCGTGCGTTACAGGATACTATTCGTGAACTGCAACGTAAGCAGGAAATCAAGTAAGGAGGCAATGTGTCGACTCAATCCAATCGTAATg                                                                                                                                                                                                                                                           <  1:608697/301‑1 (MQ=255)`  
                                                  `tcggagatgtgtataagagacagcTGGAAGGGAGCACTGATAGGATTATTTCTGATTTGCGTAGCGACAATAAGCGGTTGCGAGTCAGAGTCAAAACTACCGGAACCTCCGATGGTCAGTGTGGATTCGAGCCTGATGGTCGAGCCGAACTTGACGACCGAGATGCTAAACGTATTCTCGCAGTGACCCAGAAGGGTGACGCATGGATTCGTGCGTTACAGGATACTATTCGTGAACTGCAACGTAAGCAGGAAATCAAGTAAGGAGGCAATGTGTCTACTCAATCCAATCGTAATTCGCt                                                                                                                                                                                                                                                       <  1:451148/279‑1 (MQ=255)`  
                                                   `gagatggggataagagacagcgcTGGAAGGGGGCACTGATAGGATTATTTCTGATTTGCGTAGCGACAATAAGCGGTTGCGCGTCAGAGTCAAAACTACCGGAACCTCCGATGGTCAGTGTGGATTCGAGCCTGATGGTCGAGCCGAACTTGACGACCGAGATGCTAAACGTATTCTCGCAGTGACCCAGAAGGGTGACGCATGGATTCGTGCGTTACAGGATACTATTCGTGAACTGCAACGTAAGCAGGAAATCAAGTAAGGAGGCAATGTGTCTACTCAATCCAATCGTAATGCGCTc                                                                                                                                                                                                                                                      <  1:551219/282‑1 (MQ=255)`  
                                                   `gTATCAAGAAGACCTTGCCGCGCTGGAAGGGAGCACTGATAGGATTATTTCTGATTTGCGTAGCGACAATAATCGGTTGCGCGTCAGAGTCAAAACTACCGGAACCTCCGATGGTCAGTGTGGATTCGAGCCTGATGGTCGAGCCGAACTTGACGACCGAGATGCTAAACGTATTCTCGCAGTGACCCAGAAGGGTGACGCATGGATTCGTGCGTTACAGGATACTATTCGTGAACTGCAACGTAAGCAGGAAATCAAGTAAGGAGGCAATGTGTCTACTCAATCCAATCgctgtctctta                                                                                                                                                                                                                                                      >  1:568197/1‑291 (MQ=255)`  
                                                   `gTATCAAGAAGACCTTGCCGCGCTGGAAGGGAGCACTGATAGGATTATTTCTGATTTGCGTAGCGACAATAAGCGGTTGCGCGTCAGAGTCAAAACTACCGGAACCTCCGATGGTCAGTGTGGATTCGAGCCTGATGGTCGAGCCGAACTTGACGACCGAGATGCTAAACGTATTCTCGCAGTGACCCAGAAGGGTGACGCATGGATTCGTGCGTTACAGGATACTATTCGTGAACTGCAACGTAAGCAGGAAATCAAGTAAGGAGGCAATGTGTCTACTCAATCCAATCGTAATGCGCTc                                                                                                                                                                                                                                                      >  1:365145/1‑301 (MQ=255)`  
                                                   `gTATCAAGAAGACCTTGCCGCGCTGGAAGGGAGCACTGATAGGATTATTTCTGATTTGCGTAGCGACAATAAGCGGTTGCGCGTCAGAGTCAAAACTACCGGAACCTCCGATGGCCAGTGTGGATTCGAGCCTGATGGTCGAGCCGAACTTGACGACCGAGATGCTAAACGTATTCTCGCAGTGACCCAGAAGGGTGACGCATGGATTCGTGCGTTACAGGATACTATTCGTGAACTGCAACGTAAGCATGAAATCAAGTAAGGAGGCAATGTGTCTACTcctgtctcttatacacatctc                                                                                                                                                                                                                                                      >  1:344690/1‑281 (MQ=255)`  
                                                       `cAAGAAGACCTTGCCGCGCTGGAAGGGAGCACTGATAGGATTATTTCTGATTTGCGTAGCGACAATAAGCGGTTGCGCGTCAGAGTCAAAACTACCGGAACCTCCGATGGTCAGTGTGGATTCGAGCCTGATGGTCGAGCCGAACTTGACGACCGAGATGCTAAACGTATTCTCGCAGTGACCCAGAAGGGTGACGCATGGATTCGTGCGTTACTGGATACTATTCGTGAACTGCAACGTAAGCAGGAAATCAAGTAAGGAGGCAATGTGTCTACTcctgtctcttatacacatctccgag                                                                                                                                                                                                                                                  >  1:95202/1‑277 (MQ=255)`  
                                                       `aaagaagACCTTGCCGCGCTGGAAGGGAGCGCTGATAGGATGATTTCTGATTTGCGTAGCGAAAATAAGCAGTTGCGCGTCAGAGTAAAAACTACCGGAACCTCCGATGGTCAGTGTGGATTCGAGCCTGATGATCGAGCCGAACTTGACGACCGAGATGCTAAACGTATTCTCGCAGTGACCCAGAAGGGTGACGCATGGATTCGTGCGTTACAGGATACTATTCGTGAACTGCAACGTAAGCAGGAAATCAAGTAAGGAGGCAATGTGTCTACTCAATCCAATCGTAATGCGCTCGTAg                                                                                                                                                                                                                                                  <  1:433080/300‑1 (MQ=255)`  
                                                           `aagaCCTTGCCGCGCTGGAAGGGAGCACTGATAGGATTATTTCTGATTTGCGTAGCGACAATAAGCGGTTGCGCGTCAGAGTCAAAACTACCGGAACCTCCGATGGTAAGTGTGGATTCGAGCCTGATGGTCGAGCCGAACATGACGACCGAGATGCTAAACGTATTCTCGCAGTGACCCAGAAGGGTGACGCATGGATTCGTGCGTTACAGGATACTATTCGTGAACTGCAACGTAAGCAGGAAATCAAGTAAGGAGGCAATGTGTCTACTCAATCCAATCGTAATGCGCTCGTAGTGgc                                                                                                                                                                                                                                              <  1:62776/301‑1 (MQ=255)`  
                                                                     `cgcgtgggAAGGGAGCACTGATAGGATTATTGCTGATTTGCGTAGAGACAATAAGCGGTTGCGCGTCAGAGTCAAAACTACCGGAACCTCCGATGGTCAGTGTGGATTCGAGCCTGATGGTCGAGCCGAACTTGACGACCGAGATGCTAAACGTATTCTCGCAGTGACCCAGAATGGTGACGCATGGATTCGTGCGTTACAGGATACTATTCGTGAACTGCAACGTAAGCAGGAAATCAAGTAAGGAGGCAATGTGTCTACTCAATCCAATCGTAATGCGCTCGTAGTGGCGCAACTGaaa                                                                                                                                                                                                                                    <  1:481394/295‑1 (MQ=255)`  
                                                                      `gcgcTGGAAGGGAGCACTGATAGGATTATTTCTGATTTGCGTAGCGACAATAAGCGGTTGCGCGTCAGAGTCAAAACTACCGGAACCTCCGATGGTCAGTGTGGATTCGAGCCTGATGGTCGAGCCGAACTTGACGACCGAGATGCTAAACGTATTCTCGCAGTGACCCAGAAGGGTGACGCATGGATGCGTGCGTTACAGGATACTATTCGTGAACTGCAACGTAAGCAGGAAATCAAGTAAGGAGGCAATGTGTCTACTCAATACAATCGTAATGCGATCGTAGTGGCGCAACTGAAAg                                                                                                                                                                                                                                   >  1:258027/1‑301 (MQ=255)`  
                                                                         `cTGGAAGGGAGCACTGATAGGATTATTTCTGATTTGCGTAGCGACAATAAGCGGTTGCGCGTCAGAGTCAAAACTACCGGAACCTCCGATGGTCAGTGTGGATTCGAGCCTGATGGTCGAGCCGAACTTGACGACCGAGATGCTAAACGTATTCTCGCAGTGACCCAGAAGGGTGACGCATGGATTCGTGCGTTACAGGATACTATTCGTGAACTGCAACGTAAGCAGGAAATCAAGTAAGGAGGCAATGTGTCTACTCAATCCAATCGTAATGCGCTCGTAGTTGCGCAACTGAAAGgag                                                                                                                                                                                                                                >  1:389008/1‑301 (MQ=255)`  
                                                                           `ggAAGGGAGCACTGATAGGATTATTTCTGATTTGCGTAGCGACAATAAGCGGTTGCGCGTCAGAGTCAAAACTACCGGAACCTCCGATGGTCAGTGTGGATTCGAGCCTGATGGTCGAGCCGAACTTGACGACCGAGATGCTAAACGTATTCTCGCAGTGACCTAGAAGGGTGACGCATGGATTCGTGCGTTACAGGATACTATTCGTGAACTGCAACGTAAGCAGGAAATCAAGTAAGGAGGCAATGTGTCTACTCAATCCAATCGTAATGCGCTCGTAGTGGCGCAACTGAAAGGAGAc                                                                                                                                                                                                                              <  1:291101/301‑1 (MQ=255)`  
                                                                                  `agaaCTGATAGGATTATTTCTGATTTGCGTAGCGACAATAAGCGGTTGCGCGTCAGAGTCAAAACTACCGGAACCTCCGATGGTCAGTGTGGATTCGAGCCTGATGGTCGAGCCGAACTTGACGACCGAGATGCTAAACGTATTCTCGCAGTGACCCAGAAGGGTGACGCATGGATTCGTGCGTTACAGGATACTATTCGTGAACTGCAACGTAAGCAGGAAATCAAGTAAGGAGGCAATGTGTCTACTCAATCCAATCGTAATGCGCTCGTAGTGGCGCAACTGAAAGGAGACTTCGTgg                                                                                                                                                                                                                       <  1:639666/298‑1 (MQ=255)`  
                                                                                    `aaatGATAGGATTATTTCTGATTTGCGTAGCGACAATAAGCGGTTGCGCGTCAGAGTCAAAACTACCGGAACCTCCGATGGTGAGTGTGGATTCGAGCCTGATGGTCGAGCCGAACTTGACGACCGAGATGCTAAACGTATTCTCGCAGTGAACCAGAAGGGTGACGCATGGATTCGTGCGTTACAGGATACTATTCGTGAACTGCAACGTAAGCAGGAAATCAAGTAAGGAGGCAATGTGTCTACTCAATCCAATCGTAATGCGCTCGTAGTGGCGCAACTGAAAGGAGACTTCGTGGCg                                                                                                                                                                                                                     <  1:494052/298‑1 (MQ=255)`  
                                                                                      `cTGATAGGATTATTTCTGATTTGCGTAGCGACAATAAGCGGTTGCGCGTCAGAGTCAAAACTACCGGAACCTCCGATGGTCAGTGTGGATTCGAGCCTGATGGTCGAGCCGAACTTGACGACCGAGATGCTAAACGTATTCTCGCAGTGACCCAGAAGGGTGACGCATGGATTCGTGCGTTACAGGATACTATACGTGAACTGCAACGTAAGCAGGAAATCAAGTAAGGAGGCAATGTGTCTACTCAATCCAATCGTAATGCGCTCGTAGTGGCGCAACTGAAAGGAGACTTCGTGGCggt                                                                                                                                                                                                                   >  1:377766/1‑299 (MQ=255)`  
                                                                                          `tAGGATTATTTCTGATTTGCGTAGGGACAATAAGCGGTTGCGCGTCAGAGTCAAAACTACCGGAACCTCCGATGGTCAGGGTGGATTCGAGCCTGATGGTCGAGCCGAACTTGACGACCGAGATGCTAAACGTATTCTCGCAGTGACCCAGAAGGGTGACGCATGGATTCGTGCGTTACAGGATACTATTCGTGAACTGCAACGTAAGCAGGAAATCAAGTAAGGAGGCAATGTGTCTACTCAATCCAATCGTAATGCGCTCGTAGTGGCGCAACTGAAAGGAGACTTCGTGGCGTTCCTa                                                                                                                                                                                                               <  1:413378/301‑1 (MQ=255)`  
                                                                                            `ggATTATTTCTGATTTGCGTAGCGACAATAAGCGGTTGCGCGTCAGAGTCAAAACTACCGGAACCTCCGATGGTCAGTGTGGATTCGAGCCTGATGGTCGAGCCGAACTTGACGACCGAGAGGCTAAACGTATTCTCGCAGTGACCCAGAAGGGTGACGCATGGATTCGTGCGTTACAGGATAATATTCGGGAACTGCAACGTAAGCAGGAAATCAAGTAAGGAAGCAATGTGTCTACTCAATCCAATCGTAATGCGATCGTAGTGGCGCAAATGAAAGGAGAATTCGTGGCGTTCCTCtt                                                                                                                                                                                                             >  1:321248/1‑301 (MQ=255)`  
                                                                                             `tattactTCTGATTTGGGTCGCGCCGAGAGGCGGTTGCTCGTCAGAGTGAAAACTACCGGAACCTCCGGTGGGCCTGTTTGATTCTAGACTGATGGTCGAGCCGAATTTGACGACCGAGTTGCTACACGTATTCTCGCAGTGACCCAGCAGGTTGCCTCATGGATTCGTGCGTTACAGGATTCTATCCGTGAACTGCAACGTAAGCAGGATATCCAGTAAGGATGCACTGTGTCTACTCAATCCAATCGTAATGCTCTCGTAGTGGCGCAACTGAAAGGAGACTTCGTGGCGTTCCTATTc                                                                                                                                                                                                            <  1:658752/300‑1 (MQ=255)`  
                                                                                             `gATTATTTCTGATTTGCGTAGCGACAATAAGCGGTTGCGCGTCAGAGTCAAAACTACCGGAACCTCCGATGGTCAGTGTGGATTCGAGCCTGATGGTCGAGCCGAACTTGACGACCGAGATGCTAAACGTATTCTCGCAGTGACCCAGAAGGGTGACGCATGGATTCGTGCGTTACAGGATACTATTCGTGAACTGCAACGTAAGCAGGAAATCAAATAAGGAGGCACTGTGTCTACTCAATCCAATCGTAATTCGCTCGTAGTGGCGCAACTGAACGGAGACTTCTTGGCGTTCCTAttt                                                                                                                                                                                                            >  1:499307/1‑300 (MQ=255)`  
                                                                                             `gATTATTTCTGATTTGCGTAGCGACAATAAGCGGGTGCGCGTCAGAGTCAAAACTACCGGAACCTCCGATGGTCAGTGTGGATTCGAGCCTGATGGTCGAGCCGAACTTGACGACCGAGATGCTAAACGTATTCTCGCAGTGACCCAGAAGGGTGACGCATGGATTCGTGCGTTACAGGATACTATTCGTGAACTGCAACGTAAGCAGGAAATCAAGTAAGGAGGCAATGTGTCTACTCAATCCAATCGTAATGCGCTCGTAGTGGCGCAACTGAAAGGAGACTTCGTGGcctgtctcttt                                                                                                                                                                                                            >  1:475725/1‑291 (MQ=255)`  
                                                                                                   `ttCTGATTTGCGTAGCGACAATAAGCGGTTGCGAGTCAGAGTCAAAACTACCGGAACCTCCGATGGTCAGTGTGGATTCGAGCCTGATGGTCGAGCCGAACTTGACGACCGAGATGCTAAACGTATTCTCGCAGTGACCCAGAAGGGTGACGCATGGATTCGTGCGTTACAGGATACTATTCGTGAACTGCAACGTAAGCAGGAAATCAAGTAAGGAGGCAATGTGTCTACTCAATCCAATCGTAATGCGCTCGTAGTGGCGCAACTGAAAGGAGACTTCGTGGCGTTCCTATTCGTCTTa                                                                                                                                                                                                      <  1:177173/301‑1 (MQ=255)`  
                                                                                                      `tGATGTGCGTAGCGACAATAAGCGGTTGCGCGTCAGAGTCAAAACTACCGGAACCTCCGATGGTCAGTGTGGATTCGAGCCTGATGGTCGAGCCGAACTTGACGACCGAGATGCTAAACGTATTCTCGCAGTGACCCAGAAGGGTGACGCATGGATTCGTGCGTTACAGGATACTATTCGTGAACTGCAACGTAAGCAGTAAATCAAGTAAGGAGGCAATGTGTCTACTCAATCCAATCGTAATGCGCTCGTAGTGGCGCAACTGAAAGGAGACTTCGTGGCGTTCCTATTCGTCTTATgg                                                                                                                                                                                                   <  1:60249/301‑1 (MQ=255)`  
                                                                                                       `gagttGCGTAGCGACAATAAGCGGTTGCGCGTCAGAGTCAAAACTACCGGAACCTCCGAGGGTCAGTGTGGATTCGAGCCTGATGGTCGAGCCGAACTTGACGACCGAGATGCTAAACGTATTCTCGCAGTGACCCAGAAGGGTGACGCATGGATTCGTGCGTTACAGGATACTATTCGTGAACTGCAACGTAAGCAGGAAATCAAGTAAGGAGGCAATGTGTCTACTCAATCCAATCGTAATGCGCTCGTAGTGGCGCAACTGAAAGGAGACTTCGTGGCGTTCCTATTCGTCTTATGGa                                                                                                                                                                                                  <  1:332419/298‑1 (MQ=255)`  
                                                                                                                  `cagcAATAAGCGGTTGCGCGTCAGAGTCAAAACTACCGGAACCGCCGATGGTCAGTGTGGATTCGAGCCTGATGGTCGAGCCGAACTTGACGACCGAGATGCTAAACGTATTCTCGCAGTGACCCAGAAGGGTTACGCATGGATTCGTGCGTTACAGGATACTATTCGTGAACTGCAACGTAAGCAGGAAATCAAGTAAGGAGGCAATGTGTCTACTCAATCCAATCGTAATGCGCTCGTAGTGGCGCAACTGAAAGGAGACTTCGTGGCGTTCCTATTCGTCTTATGGAAGGCGCTAAAc                                                                                                                                                                                       <  1:420309/298‑1 (MQ=255)`  
                                                                                                                                 `gcgcGTTAGAGTCAAAACTACCGGAACCTCCGATGGTCAGTGTGGATTCGAGCCTGATGGTCGAGCCGAACTTGACGACCGAGATGCTAAACGTATTCTCGCAGTGACCCAGAAGGGTGACGCATGGATTCGTGCGTTACAGGATACTATTCGTGAACTGCAACGTAAGCAGGAAATCAAGTAAGGAGGCAATGTGTCTACTCAATCCAATCGTAATGCGCTCGTAGTGGCGCAACTGAAAGGAGACTTCGTGGCGTTCCTATTCGTCTTATGGAAGGCGCTAAACCTACCGGTGCCCACt                                                                                                                                                                        <  1:589789/301‑1 (MQ=255)`  
                                                                                                                                      `tCAGAGTCAAAACTACCGGAACCTCCGATGGTCAGTGTGGATTCGAGCCGGATGGTCGAGCCGAACTTGACGACCGAGATGCTAAACGTATTCTCGCAGTGACCCAGAAGGGTGACGCATGGATTCGTGCGTTACAGGATACTATTCGTGAACTGCAACGTAAGCAGGAAATCAAGTAAGGAGGCAATGTGTCTACTCAATCCAATCGTAATGCGCTGGTAGTGGCGCAACTGAAAGGAGACTTCGTGGCGTTCCTATTCGTCTTATGGAAGGCGCTAAACCTACCGGTGCCCACTAAgtg                                                                                                                                                                   <  1:246159/301‑1 (MQ=255)`  
                                                                                                                                         `gagTCAAAACTACCGGAACCTCCGATGGTCAGTGTGGATTCGAGCCTGATGGTCGAGCCGAACTAGACGACCGAGATGCTAAACGTATTCTCGCAGTGACCCAGAAGGGTGACGCATGGATTCGTGCGCTACAGGATACTATTAGTGAACTGCAACGTAAGCAGGAAATCAAGTAAGGAGGCAATGTGTCTACTCAATCCAATCGTAATGCGCTCGTAGTGGCGCAACTGAAAGGAGACTTCGTGGCGATCCAATTCATCTTATGGAAGGCACAAAACCTACCGGTGCCCACTAAgacagg                                                                                                                                                                >  1:646167/1‑296 (MQ=255)`  
                                                                                                                                            `tCAAAACTACCGGAACCTCGGATGGTCAGTGTGGATTCGGGCCTGATGGTCGAGCCGAACTTGACGACCGAGATGCTAAACGTATTCTCGCAGTGACCCAGAAGGGTGACGCATGGATTCGTGCGTTACAGGATACTATTCGTGAACTGCAACGTAAGCAGGAAATCAAGTAAGGAGGCAATGTGTCTACTCAATCCAATCGTAATGCGCACGTAGTGGCGCAACTGAAAGGAGACTTCGTGGCGTTCCTAGTCGTCTTATGGAAGGCGCTAAACCTACCGGTGCCCACTAAGTGTCAGAt                                                                                                                                                             <  1:252479/301‑1 (MQ=255)`  
                                                                                                                                            `acAAAACTACAGGAACCGCCGATGGTCAGTGTGGATTCGAGCCTGATGGTCGAGCCGAACTTGACGACCGAGATGCTAAACGTATTCTCGCAGTGACCCAGAAGGGTGACGCATGGATTCGTGCGTTACAGGATACTATTCGTGAACTGCAACGTAAGCAGGAAATCAAGTAAGGAGGCAATGTGTCTACTCAATCCAATCGTAATGCGCTCGTAGTGGCGCAACTGAAAGGAGACTTCGTGGCGTTCCTATTCGTCTTATGGAAGGCGCTAAACCTACCGGTGCCCACTAAGTGTCAGAt                                                                                                                                                             <  1:665659/300‑1 (MQ=255)`  
                                                                                                                                               `aaaCTACCGGAACCTCCGATGGTCAGTGTGGATTCGAGCCTGATGGTCGAGCCGAACTTGACGACCGAGATGCTAAACGTATTCTCGCAGTGACCCAGAAGGGTGACGCATGGATTCGTGCGTTACAGGATACTATTCGTGAACTGCAACGTAAGCAGGAAATCAAGTAAGGAGGCAATGTGTCTACTCAATCCAATCGTAATGCGCTCGTAGTGGCGCAACTGAAAGGAGACTTCGTGGCGTTCCTATTCGTCTTATGGAAGGCGCTAAACCTACCGGTGCCCACTAAGTGTCAGATTGa                                                                                                                                                          >  1:209003/1‑301 (MQ=255)`  
                                                                                                                                                  `cTACCGGAACCTCCGATGGTCAGTGTGGATTCGAGCCTGATGGTCGAGCCGAACTTGACGACCGAGATGCTAAACGTATTCTCGCAGTGACCCAGAAGGGTGACGCATGGATTCGTGCGTTACAGGATACTATTCGTGAACTGCAACGTAAGCAGGAAATCAAGTAAGGAGGCAATGTGTCTACTCAATCCAATCGTAATGCGCTCGTAGTGGCGCAACTGAAAGGAGACTTCGTTGCGTTCCTATTCGTCTTATGGAAGGCGCTAAACCTACCGGTGCCCACTAAGTGTCAGATTGACAt                                                                                                                                                       >  1:107170/1‑301 (MQ=255)`  
                                                                                                                                                      `cGGAACCTCCGATGGTCAGTGTGGATTCGAGCCTGATGGTCGAGCCGAACTTGACGACCGAGATGCTAAACGTATTCTCGCAGTGACCCAGAAGGGTGACGCATGGATTCGTGCGTTACAGGATACTATTCGTGAACTGCAACGTAAGCAGGAAATCAAGTAAGGAGGCAATGTGTCTACTCAATCCAATCGTAATGCGCTCGTAGTGGCTCAACTGAAAGGAGACTTCGTGGCGTTCCTATTCGTCTTATGGAAGGCGCTAAACCTACCGGTGCCCACTAAGTGTCAGATTGACATGGCt                                                                                                                                                   <  1:396298/301‑1 (MQ=255)`  
                                                                                                                                                           `ccTCCGATGGTCAGTGTGGATTCGAGCCTGATGGTCGAGCCGAACTTGACGACCGAGATGCTAAACGTATTCTCGCAGTGACCCAGAAGGGTGACGCATGGATTCGTGCGTTACAGGATACTATTCGTGAACTGCAACGTAAGCAGGAAATCAAGTAAGGAGGCAATGTGTCTACTCAATCCAATCGTAATGCGCTCGTAGTGGCGCAACTGAAAGGAGACTTCGTGGCGTTCCTATTCGTCTTATGGAAGGCGCTAAACCTACCGGTGCCCACTAAGTGTCAGATTGACATGGCTAAGGt                                                                                                                                              <  1:523213/301‑1 (MQ=255)`  
                                                                                                                                                            `cTCCGATGGTCAGTGTGGATTCGAGCCTGATGGTCGAGCCGAACTTGACGACCGAGATGCTAAACGTATTCTCGCAGTGACCCAGAAGGGTGACGCATGGATTCGTGCGTTACAGGATACTATTCGTGAACTGCAACGTAAGCAGGAAATCAAGTAAGGAGGCAATGTGTCTACTCAATCCAATCGTAATGCGCTCGTAGTGGCGCAACTGAAAGGAGACTTCGTGGCGTTCCTATTCGTCTTATGGAAGGCGCTAAACCTACCGGTGCCCACTAAGTGTCAGATTGACATGGCTAAGGTg                                                                                                                                             >  1:67223/1‑301 (MQ=255)`  
                                                                                                                                                            `cTCCGATGGTCAGTGTGGATTCGAGCCTGATGGTCGAGCCGAACTTGACGACCGAGATGCTAAACGTATTCTCGCAGTGACCCAGAAGGGTGACGCATGGATTCGTGCGTTACAGGATACTATTCGTGAACTGCAACGTAAGCAGGAAATCAAGTAAGGAGGCAATGTGTCTACTCAATCCAATCGTAATGCGATCGTAGTGGCGCAACTGAAAGGAGACTTCGTGGCGTTCCTATTCGTCTTATGGAAGGCGCTAAACCTACCGGTGCCTACTCAGTGTCAGATTGACATGGCTAAggcg                                                                                                                                             >  1:440921/1‑299 (MQ=255)`  
                                                                                                                                                              `ccGATGGTCAGTGTGGATTCGAGCCTGATGGTCGAGCCGAACTTGACGACCGAGATGCTAAACGTATTCTCGCAGTGACCCAGAAGGGTGACGCATGGATTCGTGCGTTACAGGATACTATTCGTGAACTGCAACGTAAGCAGGAAATCAAGTAAGGAGGCAATGTGTCTACTCAATCCAATCGTAATGCGCTCGTAGTGGCGCAACTGAAAGGAGACTTCGTTGCGTTCCTATTCGTCTTATGGAAGGCGCTAAACCTACCGGTGCCCACTAAGTGTCAGATTGACATGGCTACGGTGCt                                                                                                                                           >  1:594767/1‑301 (MQ=255)`  
                                                                                                                                                              `ccGATGGTCAGTGTGGATTCGAGCCTGATGGTCGAGCCGAACTTGACGACCGAGATGCTAAACGTATTCTCGCAGTGACCCAGAAGGGTGACGCATGGATTCGTGCGTTACAGGATACTATTCGTGAACTGCAACGTAAGCAGGAAATCAAGTAAGGAGGAAATGTGTCTACTCAATCCAATCGTAATGCGCTCGTAGTGGCGCAACTGAAAGGAGACTTCGTGGCGTTCCTATTCTTCTTATGGAAGGCGCTAAACCTACCGGTGCCCACTACTTGTCAGATTGACATGGCTAAGGTGCt                                                                                                                                           >  1:172525/1‑301 (MQ=255)`  
                                                                                                                                                                   `ggTCAGTGTGGATTCGAGCCTGATGGTCGAGCCGAACTTGACGACCGAGATGCTAAACGTATTCTCGCAGTGACCCAGAAGGGTGACGCATGGATTCGTGCGTTACAGGATACTATTCGTGAACTGCAACGTAAGCAGGAAATCAAGTAAGGAGGCAATGTGTCTACTCAATCCAATCGTAATGCGCTCGTAGTGGCGCAACTGAAAGGAGACTTCGTGGCGTTCCTATTCGTCTTATGGAAGGCGCTAAACCTACCGGTGCCCACTAAGTGTCAGATTGACATGGCTAAGGTGCTGGCGa                                                                                                                                      >  1:227273/1‑301 (MQ=255)`  
                                                                                                                                                                     `gcAGTGTGGATTCGGGCCTGATGGTCGAGCCGAACTTGACGACCGAGATGCTAAACGTATTCTCGCAGTGACCCAGAAGGGTGACGCATGGATTCGTGCGTTACAGGATACTATTCGTGAACTGCAACGTAAGCAGGAAATCAAGTAAGGAGGCAATGTGTCTACTCAATCCAATCGTAATGCGCTCGTAGTGGCGCAACTGAAAGGAGACTTCGTGGCGTTCCTATTCGTCTTATGGAAGGCGCTAAACCTACCGGTGCCCACTAAGTGTCAGATTGACATGGCTAAGGTGCTGGCGAAt                                                                                                                                    <  1:128498/300‑1 (MQ=255)`  
                                                                                                                                                                       `aGTGTTGATTCGAGGCGAATGGTCGAGCCGAAATTGACGACCGAGATGCTAAACGTATTCTAGAAGGGACCCAGAAGGGTGAGGCATGGATTCGTGCGTTACAGGAGACTATTCGTGAACTGCAACGTAAGCAGGAAATCAAGTAAGGAGGCAATGTGTCTACTCAATCCAATCGTAATGCGCTCGTAGTGGAGCAACTGAAAGGAGACTTCGTGGCGTTCCTATTCGTCTTATGGAAGGCGCTAAACCTACCGGTGCCCACTAAGTGTCAGATTGAAATGGCTAAGGTGCTGGCGAATgg                                                                                                                                  <  1:407362/301‑1 (MQ=255)`  
                                                                                                                                                                       `aGTGTGGATTCGAGCCTGATGGTCGAGCCGAACTTGACGACCTAGATGCTAAACCTATTCTCGCAGTGACCCAGAAAGGTGACGCATGGATTCGTGCGTTACAGGATACTATTCGTGAACTGCAACGTAAGCAGGAAATCAAGTCAGGAGGCAATGTGTCTACTCAATCCAAACGTAATGCGCTCGTAGTGGCGCAACTGAAAGGAGACTTCGTGGCGTTCCTATTCGTCTTATGGAAGGCGCTAAACCTACCGGTGCCCACTAAGTGTCAGATTGACATGGCTAAGGTGCTGGCGAATgg                                                                                                                                  <  1:30464/301‑1 (MQ=255)`  
                                                                                                                                                                        `gtgtGGATTCGAGCCTGATGGTCGAGCCGAACTTGACGACCGAGATGCTAAACGTATTCTCGCAGTGACCCAGAAGGGTAACGCATGGATTCGTGCGTTACAGGATACTATTCGTGAACTGCAACGTAAGCAGGAAATCAAGTAAGGAGGCAATGTGTCTACTCAATCCAATCGTAATGCGCTCGTAGTGGCGCAACTGAAAGGAGACTTCGTGGCGTTCCTATTCGTCTTATGGAAGGCGCTAAACCTACCGGTGCCCACTAAGTGTCAGATTGACATGGCTAAGGTGCTGGCGAATggg                                                                                                                                 >  1:653147/1‑300 (MQ=255)`  
                                                                                                                                                                        `gtgtGGATTCGAGCCTGATGGTCGAGCCGAACTTGACGACCGAGATGCTAAACGTATTCTCGAAGTGACCCAGAAGGGTGACGCATGGATTCGTGCGTTACAGGATACTATTCGTGAACTGCAACGTAAGCAGGAAATCAAGTAAGGAGGCAATGTGTCTACTCAATCCAATCGTAATGCGCTCGTAGTGGAGCAACTGAAAGGAGACTTCGTGGCGTTCCTATTCGTCTTATGGAAGGCGCTAAACCTACCGGTGCCCACTAAGTGTCAGATTGACATGGCtctgtctcttatacacatc                                                                                                                                 >  1:138706/1‑283 (MQ=255)`  
                                                                                                                                                                        `gtgtGGATTCGAGCCTGATGGTCGAGCCGAACTTGACGACCGAGATGCTAAACGTATTATCGCAGTGACCCAGAAGGGTGACGCATGGATTCGTGCGTTACAGGATACTATTCGTGAACTGCAACGTAAGCAGGAAATCAAGTAAGGATGCAATGTGTCTACTCAATCCAATCGTAATGCGCTCGTAGTGGCGCAACTGAAAGGAGACTTCGTGGCGTTCCTATTCGTCTTATGGAAGGCGCTAAACCTACCGGTGCCCACTAAGTGTCAGATTGACATGGCTAAGCTGCTGCCGACTggg                                                                                                                                 >  1:556963/1‑300 (MQ=255)`  
                                                                                                                                                                                            `gTCGAGCCGAAATTGACGACCGAGATGCTAAACGTATTCTCGCAGTGACCCAGAATGGTGACGCATGGATTCGTGCGATACAGGATAATAGTCGTGAACTGCAACGTAAGCAGGAAATCAAGTAAGGAGGCAATGTGTCTACTCAATCCAAGCGTAATGCGCTCGTAGTGGCGCAACTGAAAGGAGACTTCGTGGCGTTCCTATTCGTCTTATGGAAGGAGCTAAACCTACCGGTGCCCACTAAGTGTCAGATTGACATGGCTAAGGTGCTGGCGAATGGAGACAACAAGAAGTTCATCtt                                                                                                             <  1:265379/301‑1 (MQ=255)`  
                                                                                                                                                                                            `agagacaggAACTTGACGACCGAGATTCTAAACGTATGCTCGCAGTGACCCAGAAGGATGACGCATGGATTCGTGCGTTACAGGATACTATTCGTGAACTGCAACGTAAGCAGGAAATCAAGTAAGGAGGCAATGTGTCTACGCAATCCAAGCGTAATGCGCTCGTAGTGGCGCAACTGAAAGGAGACTTCGTGGCGTTCCTATTCGTCTTATGGAAGGCGCTAAACCTACCGGTGCCCACTAAGTGTCAGATTGACATGGCTAAGGTGCTGGCGAATGGAGACAACAAGAAGTTCATCtt                                                                                                             <  1:238829/293‑1 (MQ=255)`  
                                                                                                                                                                                                       `cTTGACGACCGAGATGCTAAACGTATTCTCGCAGTGACCCAGAAGGGTGACGCATGGATTCGTGCGTTACAGGATACTATTCGTGAACTGCAACGTAAGCAGGAAATCAAGTAAGGAGGCAATGTGTCTACTCAATCCAATCGTAATGCGCTCGTAGTGGCGCAACTGAAAGGAGACTTCGTGGGGTTCCTATTCGTCTTATGGAAGGCGCTAAACCTACCGGTGCCCACTAAGTGTCAGATTGACATGGCTAAGGTGCTGGCGAATGGAGACAACAAGAAGTTCATCTTACAGGCTTTcc                                                                                                  >  1:383825/1‑301 (MQ=255)`  
                                                                                                                                                                                                                   `caggCTAAACGTATTCTCGCAGTGACCCAGAAGGGTGACGCATGGATTCGTGCGTTACAGGATACTATTCGTGAACTGCAACGTAAGCAGGAAATCAAGTAAGGAGGCAATGTGTCTACTCAATCCAATCGTAATGCGCTCGTAGTGGCGCAACTGAAAGGAGACTTCGTGGCGTTCCTATTCGTCTTATGGAAGGCGCTAAACCTACCGGTGCCCACTAAGTGTCAGATTGACATGGCTAAGGTGCTGGCGAATGGAGACAACAAGAAGTTCATCTTACAGGATTTCCGTGGTATCGGTa                                                                                      <  1:574068/298‑1 (MQ=255)`  
                                                                                                                                                                                                                     `tGCTAAACGTATTCTCGCAGTGACCCAGAAGGGTGACGCATGGATTCGTGCGTTACAGGATACTATTCGTGAACTGCAACGTAAGCAGGAAATCAAGTGAGGAGGCAATGTGTCTACTCAATCCAATCGTAATGCGCTCGTAGTGGCGCAACTGAAAGGAGACTTCGTGGCGTTCCTATTCGTCTTATGGAAGGCGCTAAACCTACCGGTGCCCACTAAGTGTCAGATTGACATGGCTAAGGTGCTGGCGAATGGAGACAACAAGAAGTTctgtctcttatacacatctccgagcccacga                                                                                    >  1:83607/1‑271 (MQ=255)`  
                                                                                                                                                                                                                      `gCTAAACGTATTCTCGCAGTGACCCAGAAGGGTGACGCATGGATTCGTGCGTTACAGGATACTATTCGTGAACTGCAACGTAAGCAGGAAATCAAGTAAGGAGGCAATGTGTCTACTCAATCCAATCGTAATGCGCTCGTAGTGGCGCAACTGAAAGGAGACTTCGTGGCGTTCCTATTCGTCTTATGGAAGGCGCTAAACCTACCGGTGCCCACTAAGTGTCAGATTGACATGGCTAAGGTGCTGGCGAATGGAGACAACAAGAAGTTCATCTTACAGGCTTTCCGTGGTATCGGTAAGt                                                                                   >  1:534801/1‑301 (MQ=255)`  
                                                                                                                                                                                                                                          `gACCCAGAAGGGTGACGCATGGATTCGTGCGTTACAGGATACTATTCGTGAACTGCAACGTAAGCAGGAAATCAAGTAAGGAGGCAATGTGTCTACTCAATCCAATCGTAATGCGCTCGTAGTGGCGCAACTGAAAGGAGACTTCGTGGCGTTCCTATTCGTCTTATGGAAGGCGCTAAACCTACCGGTGCCCACTAAGTGTCAGATTGACATGGCTAAGGTGCTGGCGACTGGAGACAACAAGAAGACCATCTTACTGGCTTTCCGTGGTATCGGTAAGCCGTACAACACATGTGcggtc                                                               >  1:621806/1‑301 (MQ=255)`  
                                                                                                                                                                                                                                            `cccAGAAGGGTGACGCATGGATTCGTGCGTTACAGGATACTATTCGTGAACTGCAACGTAAGCAGGAAATCAAGTAAGGAGGCAATGTGACTACTCAATCAAATCGTAATGCGCTCGTAGTGGCGCAACTGAAAGGAGACTTCGTGGCGTTCCTATTCGTCTTATGGAAGGCGCTAAACCTACCGGTGCCCACTAAGTGTAAGATTGAAATGGCTAAGGCGCTGGCGAATGGAGACAACAAGAAGTTCATCTTACAGGCTTGCCGTGGTATCGGTAAGttgatgtctcttagtcacatctc                                                             >  1:315581/1‑279 (MQ=255)`  
                                                                                                                                                                                                                                            `cccAGAAGGGTGACGCATGGATTCGTGCGTTACAGGATACTATTCGTGAACTGCAACGTAAGCAGGAAATCAAGT‑AGGAGGCAATGTGTCTACTCAATCCAATCGTAATGCGCTCGTAGTGGCGCAACTGAAAGGAGACTTCGTGGCGTTCCTATTCGTCTTATGGAAGGCGCTAAACCTACCGGTGCCCACTAAGTGTCAGATTGACATGGCTAAGGTGCTGGCGAATGGAGACAACAAGAAGTTCATCTTACAGGCTTTCCGTGGTATCGGTAAGTCGTTCATCACATGTGcgttcgtt                                                            >  1:12190/1‑301 (MQ=255)`  
                                                                                                                                                                                                                                            `cccAGAAGGGTGACGCATGGATGCGTGCGTTACAGGATAATATTCGTGAACTGCAACGTAAGCAGGAAATCAAGTAAGGAGGCAATGTGTCTACTCAATCCAATCGTAATGCGCTCGTAGTGGCGCAACTGAAAGGAGACTTCGTGGAGTTCCTATGCGTCTTATGGAAGGCACTAAACCTACCGGTGCCCACTAAGTGTCAGATTGACATGGCTAAGGTGCTGGCGAATGGAGACAACAAGAAGTTCATCTTACAGGCTTTCCGTGGTATCGGTAAGTCGTTCATCACATGTGcgttcgt                                                             <  1:161769/301‑1 (MQ=255)`  
                                                                                                                                                                                                                                             `ccAGAAGGGTGACGCATGGATTCGTGCGTTACAGGATACTATTCGTGAACTGCAACGTAAGCAGGAAATCAAGTAAGGAGGCAATGTGTCTACTCAATCCAATCGTAATGCGCTCGTAGTGGCGCAACTGAAAGGAGACTTCGTGGCGTTCCTATTCGTCTTATGGAAGGCGCTAAACCTACCGGTGCCCACTAAGTGTCAGATTGACATGGCTAAGGTGCTGGCGAATGGAGACAACAAGAAGTTCATCTTACAGGCTTTCCGTGGTATCGGTACGTCGTTCATCACATGTGcgttcgtt                                                            >  1:540581/1‑301 (MQ=255)`  
                                                                                                                                                                                                                                                `gAAGGGTGACGCATGGATTCGTGCGTTACAGGATACTATTCGTGAACTGCAACGTAAGCAGGAAATCAAGTAAGGAGGCAATGTGTCTACTCAATCCAATCGTAATGCGCTCGTAGTGGCGCAACTGAAAGGAGACTTCGTGGCGTTCCTATTCGTCTTATGGAAGGCGCTAAACCTACCGGTGCCCACTAAGTGTCAGATTGACATGGCTAAGGTGCTGGCGAATGGAGACAACAAGAAGTTCATCTTACAGGCTTTCCGTGGTATCGGTAAGTCGttatgcctctaatacacatctccg                                                         >  1:634491/1‑279 (MQ=255)`  
                                                                                                                                                                                                                                                    `agtaagagacagATTCGTGCGTTACAGGATACTATTCGTGAACTGCAACGTAAGCAGGAAATCAAGTAAGGAGGCAATGTGTCTACTCAATCCAATCGTAATGCGCTCGTAGTGGCGCAACTGAAAGGAGACTTCGTTGCGTTCCTATTCGTCTTATGGAAGGCGCTAAACCTACCGGTGCCCACTAAGTGTCAGATTGACATGGCTAAGGTGCTGGCGAATGGAGACAACAAGAAGTTCATCTTACAGGCTTTCCGTGGTATCGGTAAGTCGTTCATCACATGTGCGTTCGTTGTGTGGt                                                     <  1:149496/290‑1 (MQ=255)`  
                                                                                                                                                                                                                                                          `gCATGGATTCGTGCGTTACAGGATACTATTCGTGAACTGCAACGTAAGCAGGAAATCAAGTAAGGAGGCAATGTGTCTACTCAATCCAATCGTAATGCGATCGTAGTGGCGCAACTGAAAGGAGACTTCGTGGCGTTCATATTCGTCTTATGGAAGGAGCTAAACCTACCGGTGCCCACTAAGTGTCAGATTGACATGGCTAAGGTGCTGGCGAATGGAGACAACAAGAAGTTCATCTTACAGGCTTTCCGTGGTATCGGTAAGTCGTTCATCACATGTGCGTTCGTTGTGTGGTCCTTAt                                               <  1:185398/301‑1 (MQ=255)`  
                                                                                                                                                                                                                                                            `ttgtataagagacagaCAGGATACTATTCGTGAACTGCAACGTAAGCAGGAAATCAAGTAAGGAGGCAATGTGTCTACTCAATCCAATCGTAATGCGCTCGTAGTGGCGCAACTGAAAGGATACTTCGTGGCGTTCCTATTCGTCTTATGGAAGGCGCTAAACTTACCGGTGCCCACTAAGTGTCAGATTGACATGGCTAAGGTGCTGGCGAATGGAGACAACAAGAAGTTCATCTTACAGGCTTTCCGTGGTATCGGTAAGTCGTTCATCACATGTGCGTTCGTTGTGTGGTCCTTATgg                                             <  1:297762/286‑1 (MQ=255)`  
                                                                                                                                                                                                                                                            `agagacaggTGCGTTACAGGATACTATTCGTGAACTGCAACGTAAGCAGGAAATCAAGTAAGGAGGCAATGTGTCTACTCAATCCAATCGTAATGCGCTCGTAGTGGCGCAACTGAAAGGAGACTTCGTGGCGTTCCTATTCGTCTTATGGAAGGCGCTAAACCTACCGGTGCCCACTAAGTGTCAGATTGACATGGCTAAGGTGCTGGCGAATGGAGACAACAAGAAGTTCATCTTACAGGCTTTCCGTGGTATCGGTAAGTCGTTCATCACATGTGCGTTCGTTGTGTGGTCCTTATgg                                             <  1:469844/293‑1 (MQ=255)`  
                                                                                                                                                                                                                                                              `ggATTCGTGCGTTACAGGATACTATTCGTGAACTGCAACGTAAGCAGGAAATCAAGTAAGGAGGCAATGTGTCTACTCAATCCAATCGTAATGCGCTCGTAGTGGCGCAACTGAAAGGAGACTTCGTGGCGTTCCTATTCGTCTTATGGAAGGCGCTAAACCTACCGGCGCCCACTAAGTGTCAGATTGACATGGCTAAGGTGCTGGCGAATGGAGACAACAAGAAGTTCATCTTACAGGCTTTCCGTGGTATCGGTAAGTCGTTCATCACATGTGCGTTCGTTGTGTGGTCCTTATGgag                                           <  1:615507/301‑1 (MQ=255)`  
                                                                                                                                                                                                                                                              `ggAGTCGTGCTTTACATGATACTATTCGTGAACTGCAACGTAAGCAGGAAATCAAGTAAGGAGGCAATGTGTCTACGCAATCCAATCGTAATGCGCTCGTAGTGGCGCAACTGAAAGGAGACTTCGTGGCGTTCCTATTCGTCTTATGGAAGGCGCTAAACCTACCGGTGCCCACTAAGTGTCAGATTGACATGGCTAAGGTGCTGGCGAATGGAGACAACAAGAAGTTCATCTTACAGGCTTTCCGTGGTATCGGTAAGTCGTTCATCACATGTGCGTTCGTTGTGTGGTCCTTATgggg                                           <  1:426518/301‑3 (MQ=255)`  
                                                                                                                                                                                                                                                               `gATTCGTGCGTTACAGGATACTATTCGTGAACTGCAACGTAAGCAGGAAATCAAGTAAGGAGGCAATGTGTCTACTCAATCCAATCGTAATGCGCTCGTAGTGGCGCAACTGAAAGGAGACTTCGTGGCGTTCCTATTCGTCTTATGGAAGGCGCTAAACCTACCGGTGCCCACTAAGTGTCAGATTGACATGGCTAAGGTGCTGGCGAATGGAGACAACAAGAAGTTCATCTTACAGGCTTTCCGTGGTATCGGTAAGTCGTTCATCACATTTGCGTTCGTTGTGTGGTCCTTATGgagc                                          >  1:156139/1‑300 (MQ=255)`  
                                                                                                                                                                                                                                                                `gagacagCGTTACAGAATACTATTCGTGAACTGCAACGTAAGCAGGAAATCAAGTAAGGAGGCAATGTGTCTACTCAGTCCAATCGTAATGCGCTCGTAGTGGCGCAACTGAAAGGAGACTTCGTGTCGTTCCTATTCGTCTTATGGAAGGCGCTAAACCTACCGGTGCCCACTAAGTGTCAGATTGACATGGCTAAGGTGCTGGCGAATGGAGACAACAAGAAGTTCATCTTACAGGCTTTCCGTGGTATCGGTAAGTCGTTCATCACATGTGCGTTCGTTGTGTGGTCCTTATGgagag                                         <  1:402136/295‑1 (MQ=255)`  
                                                                                                                                                                                                                                                                         `ttACAGGATACTATTCGTGAACTGCAACGTAAGCAGGAAATCAAGTAAGGAGGCAATGTGTCTACTCAATCCAATCGTAATGCGCTCGTAGTGGCGCAACTGAAAGGAGACTTCGTGGCGTTCCTATTCGTCTTATGGAAGGCGCTAAACCTACCGGTGCCCACTAAGTGTCAGATTGACATGGCTAAGGTGCTGGCGAATGGAGACAACAAGAAGTTCATCTTACAGGCTTTCCGTGGTAACGGTAAGTCGTTCATCACATGTTCGTTCGTTGTGTGGTCCTTATggctgacgtttatac                                >  1:89569/1‑288 (MQ=255)`  
                                                                                                                                                                                                                                                                         `ttACAGGATACTATTCGTGAACTGCAACGTAAGCAGGAAATCAAGTAAGGAGGCAATGTGTCTACTCAATCCAATCGTAATGCGCTCGTAGTGGCGCAACTGAAAGGAGACTTCGTGGCGTTCCTATTCGTCTTATGGAAGGCGCTAAACATACCGGTGCCCACTAAGTGTCAGATTGACATGGCTAAGGTGCTGGCGAATGGAGACAACAAGAAGTTCATCTTACAGGCTTTCCGTGGTATCGGTAAGTCGTTCATCACATGTGCGTTCGTTGTGTGGTCATTATGGAGAGACACTCAGt                                >  1:310007/1‑301 (MQ=255)`  
                                                                                                                                                                                                                                                                           `aCAGGATACTATTCGTGAACTGCAACGTAAGCAGGAAATCAAGTAAGGAGGCAATGTGTCTACTCAATCCAATCGTAATGCGCTCGTAGTGGCGCAACTGAAAGTAGACTTCGTGGCGTTCCTATTCGTCTTATGGAAGGCGCTAAACCTACCGGTGCCCACTAAGTGTCAGATTGACATGGCTAAGGTGCTGGCGAATGGAGACAACAAGAAGTTCATCTTACAGGCTTTCCGTGGTATCGGTAAGTCGTTCATCACATGTGCGTTCGTTGTGTGGTCCTTATGGTGAGACCCTCAGTTg                              >  1:100025/1‑301 (MQ=255)`  
                                                                                                                                                                                                                                                                           `aCAGGATACTATTCGTGAACTGCAACGTAAGCAGGAAATCAAGTAAGGAGGCAATGTGTCTACTCAATCCAATCGTAATGCGCTCGTAGTGGCGCAACTGAAAGGAGACTTCGTGGCGTTCCTATTCGTCTTATGGAAGGCGCTAAACCTACCGGTGCCCACTAAGTGTCAGATTGACATGGCTAAGGTGCTGGCGAATGGAGACAACAAGAAGTTCATCTTACAGGCTTTCCGTGGTATCGGTAAGTCGTTCATCACATGTGCGTTCGTtgctgtctcttatacacatctccgagcccac                              >  1:77543/1‑272 (MQ=255)`  
                                                                                                                                                                                                                                                                           `aCAGGATACTATTCGTGAACTGCAACGTAAGCAGGAAATCAAGTAAGGAGGCAATGTGTCTACTCAATCCAATCGTAATGCGCTCGTAGTGGCGCAACTGAAAGGAGACTTCGTGGCGTTCCTATTCGTCTTATGGAAGGCGCTAAACCTACCGGTGCCCACTAAGTGTCAGATTGACATGGCTAAGGTGCTGGCGAATGGAGACAACAAGAAGTTCATCTTACAGGCTTTCCGTGGTATCGGTAAGTCGTTCATCACATGTGCGTTCGTTGTGTGGTCCTTATGGAGAGACCCTCAGttt                              >  1:14459/1‑300 (MQ=255)`  
                                                                                                                                                                                                                                                                           `aCAGGATACTATTCGTGAACTGCAACGTAAGCAGGAAATCAAGTAAGGAGGCAATGTGTCTACTCAATCCAATCGTAATGCGCTCGTAGTGGCGCAACTGAAAGGAGACTTCGTGGCGTTCCTATTCGTCTTATGGAAGGCGCTAAACCTACCGGTGCCCACTAAGTGTCAGATTGACATGGCTAAGGTGCTGGCGAATGGAGACAACAAGAAGTTCATCTTACAGGCTTTCCGTGGTATCGGTAAGTCGTTCATCACATGTGCGTTCGTTGTGTGGTCCTTATGGAGAGACCCTCAGTTg                              >  1:277294/1‑301 (MQ=255)`  
                                                                                                                                                                                                                                                                              `ggATACTATTCGTGAACTGCAACGTAAGCAGGAAATCAAGTAAGGAGGCAATGTGTCTACTCAATCCAATCGTAATGCGCTCGTAGTGGCGAAACTGAAAGGAGACTTCGTGGCGTTCCTATTCGTCTTATGGAAGGCGCTAAACCTACCGGTGCCCACTAAGTGTCAGATTGACATGGCTAAGGTGCTGGCGAATGGAGACAACAAGAAGTTCATCTTACAGGCTTTCCGTGGTATCGGTAAGTCGTTCATCACATGTGCGTTCGTTGTGTGGTCCTTATGGAGAGACCCTCAGTTGAAg                           >  1:458233/1‑301 (MQ=255)`  
                                                                                                                                                                                                                                                                                `aTACGAGTCGTGAACTGCAACGTAAGCAGGAAAGCAAGGAAGGAGGCAATGTGTCTACTCAATCCAATCGTAATGCGCTCGTAGTGGCGCAACTGAAAGGAGACTTCGTGGCGTTCCTATTCGTCTTATGGAAGGCGCTAAACCTACCGGTGCCCACTAAGTGTCAGATTGACATGGCTAAGGTGCTGGCGAATGGAGACAACAAGAAGTTCATCTTACAGGCTTTCCGTGGTATCGGTAAGTCGTTCATCACATGTGCGTTCGTTGTGTGGTCCTTATGGAGAGACCCTCAGTTGAAGAt                         <  1:196401/301‑1 (MQ=255)`  
                                                                                                                                                                                                                                                                                   `cTATTCGTGAACTGCAACGTAAGCAGGAAATCAAGTAAGGAGGCAATGTGTCTACTCAATCCAATCGTAATGCGCTCGTAGTGGCGCAACTGAAAGGAGACTTCGTGGCGTTCCTATTCGTCTTATGGAAGGCGCTAAACCTACCGGTGCCCACTAAGTGTCAGATTGACATGGCTAAGGTGCTGGCGAATGGAGACAACAAGAAGTTCATCTTACAGGCTTTCCGTGGTATCGGTAAGTCGTTCATCACATGTTCGTTCGTTGTGTGGTCCTTATGGAGAGACCCTCAGTTGAAGATACt                      >  1:234115/1‑301 (MQ=255)`  
                                                                                                                                                                                                                                                                                     `aTTCGTGAACTGCAACGTAAGCAGGAAATCAAGTAAGGAGGCAATGTGTCTACTCAATCC‑ATCGTAATGCGCTCGTAGTGGCGCAACTGAAAGGAGACTTCGTGGCGTTCCTATTCGTCTTATGGAAGGCGCTAAACCTACCGGTGCCCACTAAGTGTCAGATTGACATGGCTAAGGTGCTGGCGAATGGAGACAACAAGAAGTTCATCTTACAGGCTTTCCGTGGTATCGGTAAGTCGTTCATCACATGTGCGTTCGTTGTTTGTTCCTTATGGAGAGACCCTCCGtctgtctcttatac                   >  1:357062/1‑288 (MQ=255)`  
                                                                                                                                                                                                                                                                                                       `aaGCAGGAAATAAAGTAAGGAGGCAATGTGTCTACTCAATCCAATCGTAATGCGCTCGTAGTGGCGCAACTGAAAGGAGACTTCGTGGCGTTCCTATTCGTCTTATGGAAGGCGCTAAACCTACCGGTGCCCACTAAGTGTCAGATTGACATGGCTAAGGTGCTGGCGAATGGAGACAACAAGAAGTTCATCTTACAGGCTTTCCGTGGTATCGGTAAGTCGTTCATCACATGTGCGTTCGTTGTGTGGTCCTTATGGAGAGACCCTCAGTTGAAGATACTTATCGTATCAGCCTCTAAgg  <  1:113298/301‑1 (MQ=255)`  

                                                                                                                                                                                                                                                                                                          `|`                                                                                                                                                                                                                                                                                                           
`AGTTGAGGCTGCGAAGAGCACTCAAAGAGCAATCGATGCGGTATCTGCTAAGTATCAAGAAGACCTTGCCGCGCTGGAAGGGAGCACTGATAGGATTATTTCTGATTTGCGTAGCGACAATAAGCGGTTGCGCGTCAGAGTCAAAACTACCGGAACCTCCGATGGTCAGTGTGGATTCGAGCCTGATGGTCGAGCCGAACTTGACGACCGAGATGCTAAACGTATTCTCGCAGTGACCCAGAAGGGTGACGCATGGATTCGTGCGTTACAGGATACTATTCGTGAACTGCAACGTAAGTAGGAAATCAAGTAAGGAGGCAATGTGTCTACTCAATCCAATCGTAATGCGCTCGTAGTGGCGCAACTGAAAGGAGACTTCGTGGCGTTCCTATTCGTCTTATGGAAGGCGCTAAACCTACCGGTGCCCACTAAGTGTCAGATTGACATGGCTAAGGTGCTGGCGAATGGAGACAACAAGAAGTTCATCTTACAGGCTTTCCGTGGTATCGGTAAGTCGTTCATCACATGTGCGTTCGTTGTGTGGTCCTTATGGAGAGACCCTCAGTTGAAGATACTTATCGTATCAGCCTCTAAGG  >  NC_001604.1/37048‑37643`

  

Alignment Legend

Aligned base mismatch/match (shaded by quality score): `ATCG/ATCG` `< 3 ≤` `ATCG/ATCG` `< 10 ≤` `ATCG/ATCG` `< 22 ≤` `ATCG/ATCG` `< 34 ≤` `ATCG/ATCG` `< 38 ≤` `ATCG/ATCG`

Unaligned base: `atcg`    Masked matching base: `atcg`    Alignment gap: `‑`    Deleted base: `‑`
